# Modeling Scanning Electrochemical Cell Microscopy (SECCM) in Twisted Bilayer Graphene

**DOI:** 10.1021/acs.jpclett.4c01002

**Published:** 2024-07-12

**Authors:** Mohammad Babar, Venkatasubramanian Viswanathan

**Affiliations:** † Department of Mechanical Engineering, University of Michigan, Ann Arbor, Michigan 48109, United States; ‡ Department of Mechanical Engineering, Carnegie Mellon University, Pittsburgh, Pennsylvania 15213, United States; § Department of Aerospace Engineering, University of Michigan, Ann Arbor, Michigan 48109, United States

## Abstract

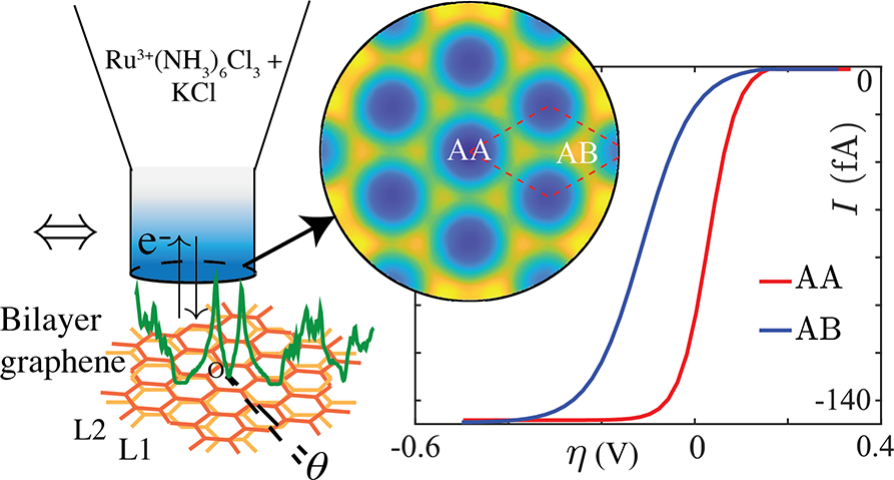

Twisted 2D-flat band materials host exotic quantum phenomena and novel moiré patterns, showing immense promise for advanced spintronic and quantum applications. Here, we evaluate the nanostructure–activity relationship in twisted bilayer graphene by modeling it under the scanning electrochemical cell microscopy setup to resolve its spatial moiré domains. We solve the steady state ion transport inside a 3D nanopipette to isolate the current response at AA and AB domains. Interfacial reaction rates are obtained from a modified Marcus–Hush–Chidsey theory combining input from a tight binding model that describes the electronic structure of bilayer graphene. High rates of redox exchange are observed at the AA domains, an effect that reduces with diminished flat bands or a larger cross-sectional area of the nanopipette. Using voltammograms, we identify an optimal voltage that maximizes the current difference between the domains. Our study lays down the framework to electrochemically capture prominent features of the band structure that arise from spatial domains and deformations in 2D flat-band materials.

Interfacial electrode–electrolyte reactions are of key importance in electrochemical devices, where they regulate the limits of operation and can enable better overall performance^1−5^ by aiding faster and more efficient charge transfer. 2D flat-band systems are a promising class of materials for this purpose due to their tunable superconductivity and correlated electronic phases.^6−10^ Significantly enhanced electron-transfer rates have been observed near the “magic” angles of twisted bilayer and trilayer graphene^11,12^ (tBLG, tTLG), almost reaching to those of bulk graphite. In these systems, flat bands at the magic angle can coincide energetically with redox couple states and thus drastically increase electron-transfer rates. Such topological twist-angle defects can help tune properties of 2D materials for robust nanoelectrochemical devices and energy storage.^11,13,14^

Twisted 2D materials also form spatial domains, e.g., AA, AB, and domain walls in tBLG,^15^ that enclose unique band structure features. To determine the electron-transfer rates, recent studies utilize nanopipettes of ∼100 nm radius over the 2D surface,^11,12^ effectively averaging the current signal from domains with much smaller length scales (∼5 nm). Zooming into these domains can provide clear evidence of novel electronic features like the flat bands inside the AA domains of tBLG.^15^ One can observe real-space signatures of density of states (DOS) artifacts like the Van Hove Singularity (VHS) peaks and separation with the twist angle. Hence, a direct correlation of the electronic structure with the moiré pattern, structural deformation, and strain fields^16^ can be established. This correlation can be utilized to study the nanostructure–activity relationship in twisted graphene and to discern its spatial features through its electrochemical response. The scanning electrochemical cell microscopy (SECCM) is based on this working principle and has been widely studied for various systems like electrocatalytic and Li-ion cathode materials, aprotic solvents, nanoparticles, corrosion at metal surfaces, etc.^13,17−23^ Scanning ion conductance microscopy (SICM)^24^ operates on a similar principle but uses a direct ionic current to produce surface charge maps and tomographic images.

To enable this development in flat-band materials, a rate model that integrates the electronic structure is necessary. This condition rules out the traditional Butler–Volmer^25,26^ and Marcus theories^27−31^ for predicting electron-transfer rates. Chidsey’s modification to Marcus theory, which adds a term with electron and hole occupations, assumes a constant, energy-independent DOS of the electrode.^1,32^ The effect from DOS is included in extended formulations of Marcus–Hush–Chidsey (MHC)^33,34^ also known as the Gerischer model.^35^ The model has been tested in a SECCM setup to compare the activity of edge vs basal sites in graphene and graphite sheets.^36^ Tuning the DOS for better overlap of the electrode states with redox couple transitions^33,37^ has explained kinetic enhancement in systems like twisted layers of graphene,^11,12^ single molecule reactions with gold and copper,^38,39^ lithium stripping and electrodeposition,^33^ and reactivity of graphene edge states and defects.^40,41^ Advances in fabrication of small pipet orifice diameters enables increased spatial resolution,^42^ thus facilitating a real-space electrochemical study of the exotic electronic properties in twisted graphene.

In this Letter, we shall use tBLG as a model system and theoretically evaluate the resolution of the SECCM setup over its spatial domains. We shall derive a steady state solution of the ion-transport equations inside the nanopipette, as has been performed to simulate and fit voltammograms with experiments.^11,12^ Using the finite element method, we solve for the electric potential and ionic concentrations self-consistently, as defined by the Poisson–Nernst–Planck (PNP) equations. These equations have been utilized to report *i*–*V* curves in other systems.^43−46^ Ionic flux and redox current are thus acquired at the orifice of the nanopipet, varying in response to the applied voltage, which alters the kinetic rates. The local electronic structure is not axi-symmetric as assumed before,^11,12^ which requires simulation in a 3D nanopipette and evaluating the rates at each polar coordinate of the orifice. We generated steady state voltammograms (SSV) by scanning the current across a range of voltages and determined the optimal driving force that yields the highest resolution of the domains. Additionally, we present a current map with the nanopipette positioned at every coordinate of the moiré unit cell. The orifice radius and twist angle, as depicted in the current map, serve as the primary parameters controlling the current resolution. We simulate nanopipettes with radii on the length scale of the tBLG domains (∼5 nm) and obtain current in femtoamperes (∼50 fA). It has been experimentally demonstrated that recording femto currents is feasible,^47−49^ and studies on ion-transfer kinetics using nanopipettes with a scale of ∼5 nm have also been conducted.^50−52^

Figure 1 shows the schematic of the SECCM setup to be used for scanning the current at a range of overpotentials, as employed earlier on bilayer and trilayer graphene systems.^11,12^ The driving voltage is applied between the solution inside the nanopipette and the substrate, and corresponding current response is recorded. The formal potential of the chosen redox couple, ruthenium hexamine (Ru^3+^(NH_3_)_6_) in aqueous KCl, is closest to the charge neutrality point (CNP) of multilayer graphene,^11,36^ allowing the most efficient capture of flat bands and AA/AB domain resolution in measured currents. In the figure, a single channel SECCM will operate in a hopping mode. As we describe later (Figure 2b), the rate model prefactors were fitted on the experimentally measured rate constants, which were collected in hopping mode.^11^ In our previous work,^11^ we ensured an overall baseline error of 50 fA in our SECCM setup before approaching or making final measurements on tBLG. The pipettes can stabilize for a minute after approaching and before measurement to reduce additional noise from the back and forth motion. To avoid overlapping between spots, in any mapping experiment, the pipette can hop for at least two pipette diameter distances between the two spots. The scan rate of hopping will depend on the wait time allowed for the pipettes to stabilize. Previously,^12^ the approach rate was set to be 0.2 μm/s and scanning potential at 100 mV/s. Drift can cause a loss in resolution, therefore the established drift correction techniques for microscopy can be employed.^53−55^

**Figure 1:**
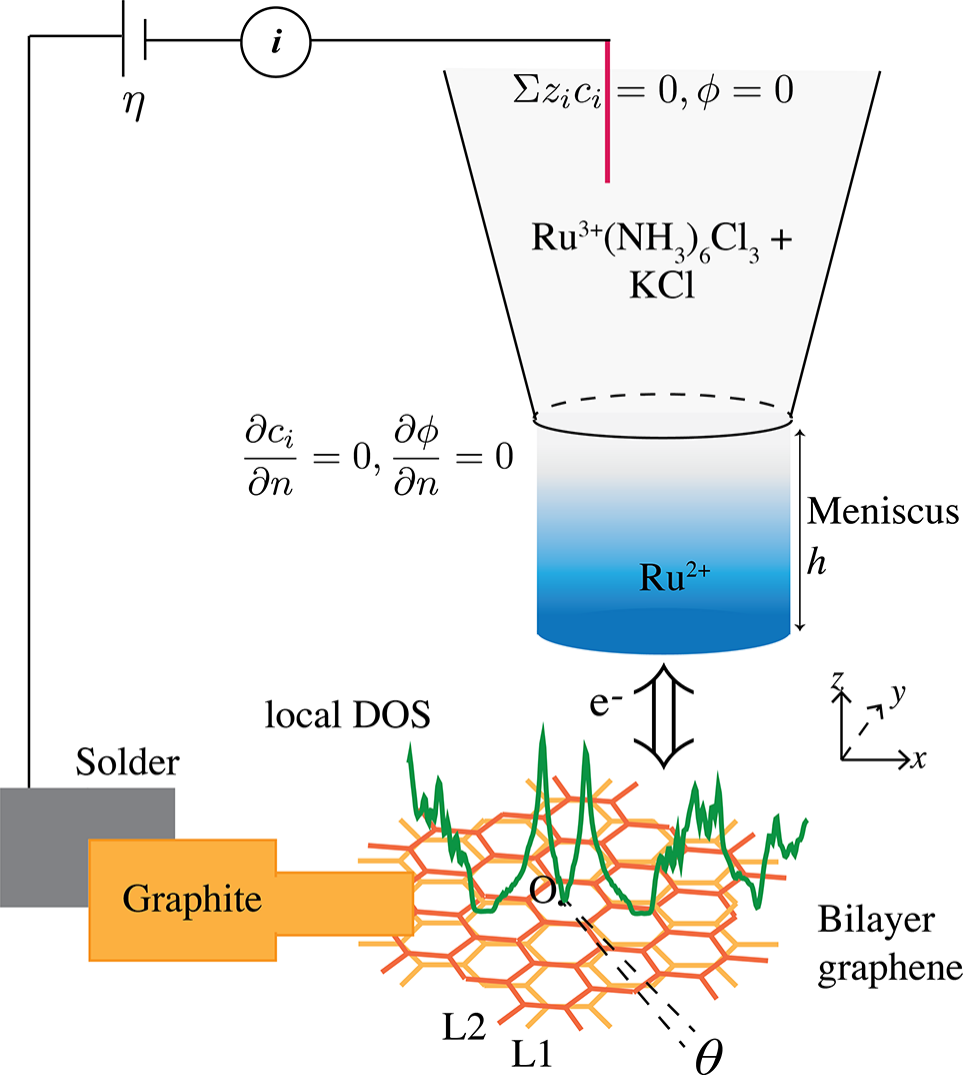
Simplified schematic of the SECCM experimental setup where the nanopipette contains the redox couple (Ru^3+^(NH_3_)_6_) and the supporting electrolyte (KCl) undergoing electron exchange with the substrate (tBLG) along the *z* axis. The top and side surfaces of the nanopipette are indicated with charge-neutrality and no flux conditions, respectively. The bottom BC is described in the main text. The nanopipette can move along the *xy* plane and capture reaction current from the locally varying DOS (green) over different tBLG domains.

**Figure 2:**
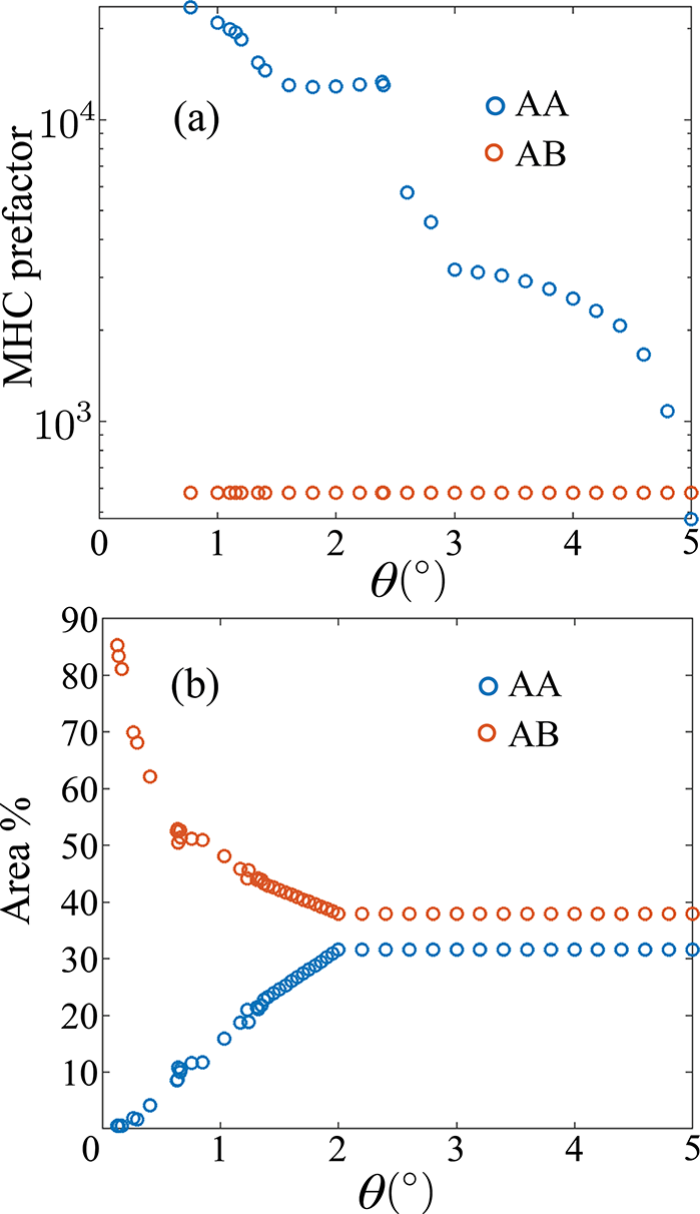
Proportionality factors for the MHC-DOS rate constants (eq 3) at AA and AB domains (a) fitted from area-weighted experimental equilibrium rate constants by Yu et al.^11^ Experimentally determined area fractions of AA and AB domains^11^ (b) as a function of twist angle θ, which plateau after 2° due to the diminished flat bands and lattice relaxation.

The previous authors^11,12^ used COMSOL^56^ to simulate the SSVs in an axi-symmetric 2D nanopipette and to fit the experimental curves. This approach is, however, limited when extending it to more sophisticated rate theories and higher dimensions. For an integral-based model like MHC, studies fit an empirical prefactor to the Butler–Volmer, before plugging it as a boundary condition (BC) in COMSOL.^11,12^ To avoid the fitting process, which may be inaccurate, we formulate the PNP system in FEniCS,^57−60^ allowing us to directly incorporate theoretical redox rates using an external Julia^61^-based package (ElectrochemicalKinetics.jl^33^). The package provides a general interface for calling popular rate models, which we utilized to parallelize the computation across real-space coordinates. Moreover, it uses adaptive Gauss–Kronrod quadrature (QuadGK.jl^62^) for accurate evaluation of integrals of peaked DOS functions found in flat-band materials.

The 2D axi-symmetric assumption is valid when the orifice radius (∼50 nm) considerably exceeds the moiré length of tBLG (13 nm at 1.1°). In this case, responses from local AA/AB domains are averaged out, and the total DOS can be used for predictions. However, local DOS is not axisymmetric. Hence, to capture the spatial domains, we switched to the 3D mesh of the nanopipette where the reaction rates are unique at each polar coordinate of the orifice. We obtained the local DOS using a low-energy tight binding model based in MATLAB.^15^ Meshes were generated using GMSH.^63^ Similar to Yu et al.,^11^ we assume the meniscus area and height to be the same as the orifice area and radius respectively, as shown in Figure 1. Both the meniscus and nanopipette volume are part of the mesh. The taper angle of the nanopipette is fixed at 10°.^11^

The current is thus independent of polar coordinates,
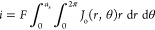
1where *F* is the Faraday’s constant, *a*_s_ is the orifice radius, and *J*_o_ is the concentration flux of the oxidized species (Ru^3+^). *J*_o_ is equal and opposite to the flux of the reduced specie (Ru^2+^), and can be related to rate constants,
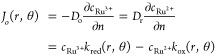
2where *c*_Ru^3+^_ and *c*_Ru^2+^_ and *D*_o_ and *D*_r_ are the concentrations and diffusion coefficients of the oxidized and reduced species, respectively. Their rate constants *k*_ox_ and *k*_red_ are derived from the selected rate theory as a function of the applied overpotential. Based on previous works,^12,64^ we set *D*_o_ and *D*_r_ of Ru^3+/2+^(NH_3_)_6_ to 8.43 × 10^–6^ cm^2^/s and 1.19 × 10^–5^ cm^2^/s, respectively. Eq 2 forms the BC at the orifice, which is the only outlet for ionic flux.

As shown previously,^11,12^ MHC-DOS theory is best suited for qualitative assessments of rate enhancement in 2D flat-band materials. Given the reorganization energy (λ), overpotential (η), temperature (*T*) and DOS  of the electrode, the rate constants are proportional to,^65^
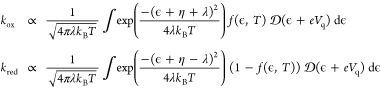
3where *f* is the Fermi–Dirac distribution and *V*_q_ is the potential across the quantum capacitance.^11,40,65^ The rest of the applied voltage drops across the electric double layer (*V*_dl_ = η – *V*_q_), which is used to formulate a Robin-BC for electric potential ϕ at the orifice, based on the Stern layer model:^66,67^
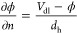
4where *d*_h_ = 0.5 nm is the constant thickness of the Helmholtz layer.^11^ The remaining BCs at the side walls (no flux, Neumann) and top surfaces (charge neutrality, Dirichlet) of the nanopipette are shown in Figure 1. Concentrations *c*_*i*_ indicate all four species (K^+^, Cl^–^, Ru^3+^, Ru^2+^) to be solved at each mesh element. To mimic the experiments,^11,12^ the unreduced Ru concentration (*c*_Ru^3+^_) is initially fixed at 2 mM at the top surface where the reduced ion (Ru^2+^(NH_3_)_6_) is absent.

A key difference from previous experiments is the concentration of the supporting electrolyte (KCl). The originally used concentration of 100 mM will induce substantial ionic current rectification (ICR) for orifice radii of ∼5 nm in this study. Previous works^42,43^ suggest that ICR is a result of the diffuse double layer (ddl) formed at the charged wall of the orifice and can be minimized by increasing the concentration of the supporting electrolyte. Specifically with quartz nanopipettes used before,^11,12^ the ICR effect is significant if the KCl concentration is ≤100 mM for ∼10 nm orifice radii.^42,44,68^ A high concentration of the supporting electrolyte also reduces ion-transport from electromigration.^69^ Hence to avoid a nonlinear *i*–*V* response, we simulate the reactions with an increased KCl concentration of 500 mM, in which case the ddl occupies only 17% of the orifice cross-section.^42^ This is the maximum value we can use without encountering numerical instabilities. If the measurements are stable, then higher concentrations may also be used in experiments.

At steady state, no convection, and finite electromigration from an electrostatic field, the ion transport is governed by the Nernst–Planck equation,
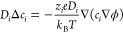
5where *z*_*i*_ is the valence of the *i*th ionic specie. Electric potential is coupled with ionic concentration through the Poisson’s equation,
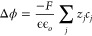
6where *T* is the room temperature, and ϵ and ϵ_o_ are relative and vacuum permittivities, respectively. As a last step, we need the real-space twist-dependent prefactors of rate constants (eq 3) to match the experimental values.

Quantitatively, the experimental rates exceed theoretical predictions from MHC-DOS by an order of magnitude.^11^ Yu et al.^11^ showed that the excessive rates are localized at the AA domains by weighing the average rate constants with area fractions of AA and AB domains (Figure 2b). For experimental accuracy of our calculations, we fit the proportionality factor in eq 3 with area-weighted equilibrium rates *k*_AA,exp_^0^ and *k*_AB,exp_^0^ shown in Figure 2a. As evident from the figure, the prefactor at AB (∼580) remains constant regardless of the twist angle. Conversely at AA, it is significantly high near the magic angle (<1.5°^11^), then decreases monotonically with twist angle due to the diminishing effect of lattice relaxation and flat bands. Eventually, it converges with the AB prefactor at a 5° twist. Figure 2b shows the AA/AB area fractions from experiments,^11^ which are used to construct the space and twist dependent prefactor field *A*(*x*, *y*, θ_*T*_) that matches the experiments. The moiré unit cell dimensions are obtained from previous studies.^70^

The applied voltage modifies the rate constants (eq 3), which on solving eqs 5 and 6 alters the Ru-ion flux (eq 2) and hence the current (eq 1) at the orifice. Sweeping the applied voltage in a positive range inhibits the current by decreasing *k*_red_/*k*_ox_ (reaction limited) until it reaches zero. Negative voltages increase current by assisting redox and Ru^2+^ accumulation, until they are capped by the mass transport limit that is dependent on the nanopipette cross-section area. Current resolution of the tBLG domains will be maximum at an intermediate voltage away from the above two limits.

An example solution of the described system can be visualized at the nanopipette orifice at two twist angles (1.1° and 4.6°, Figure S1). The radius of the nanopipet (*a*_*s*_ = 5 nm) allows a clear comparison between the two cases. Here, the nanopipette is centered at the AA domain, and the Ru^3+^ concentration (*c*_Ru^3+^_) is solved at equilibrium (η = 0 V). Depleted *c*_Ru^3+^_ indicates a greater redox and accumulation of *c*_Ru^2+^_, occurring at AA (blue spots), where flat bands localize and produce a higher rate constant.

The larger twist angle (Figure S1b) has a smaller moiré unit cell and fits entirely inside the cross-section with many visible AA and AB spots. The current resolution is weak at large angles due to two reasons: diminished flat bands at AA as evident by the minute difference in concentration between the domains (0.4%) and a small unit cell which averages out the signal over many domain spots. Consequently, SSV is almost identical when centered at the AA and AB domains (Figure S2). In contrast, the concentration difference between domains is maximum at the magic angle (23%, Figure S1a), and only one domain spot is visible in a 5 nm radius due to the large unit cell. As we show later, SSV at 1.1° over a 5 nm radius exhibits decent resolution between the AA and AB domains. Maximum current difference (Δ*I*_m_) is calculated to be 100.9 fA at −0.05 V applied voltage, i.e., 27% of its limiting current (*I*_lim_, Table 1). Current at AA exceeds that at AB in the given voltage range.

**Table 1:** Summary of Current Resolution between Domains for the Ru^3+/2+^(NH_3_)_6_ Redox Couple^a^

***a***_***s***_**(nm)**	**θ**_***tr***_**(deg)**	***r***_***c***_**(nm)**	**Δ***I*_**m**_**(fA)**	***I***_**lim**_**(fA)**	Δ*I*_m_/*I*_lim_
2	3.8	2.2	100.9	–147.2	0.68
3	2.6	3.1	109.2	–219.8	0.50
5	1.6	5.1	100.9	–368.0	0.27
8	1.0	8.2	44.2	–592.9	0.07

a At each orifice radii (*a*_s_, column 1), values for the transition angle (θ_tr_, column 2) when current maxima switch from an AA to AB center, the circumradius (*r*_c_, column 3) of the moiré basis vectors, maximum current difference Δ*I*_m_ at 1.1° twist (column 4), limiting current (*I*_lim_, column 5), and resolution ratio (Δ*I*_m_/*I*_lim_, column 6) are shown.

By scanning the nanopipette over the tBLG surface in the *xy* plane (Figure 1), current can now be calculated at each point of the moiré unit cell. The current map at two twist angles (1.1° and 2°, Figure 3) reveals an interesting phenomenon. Keeping the orifice radius fixed (5 nm, red circles), the maximum current shifts from the AA to AB center between 1.1° to 2°. In the latter case, the nanopipette centered at AB encloses three AA domains (red circle in Figure 3b), compared to a single domain in the former (Figure 3a). While centered at AA, the nanopipette only encloses one AA domain in both cases. Hence maximum current occurs at spatial points that confine most number of AA domains within the nanopipette aperture. At the transition angle (1.6°), the SSV is identical and the current is uniform over the unit cell (Figure S3). The circumradius of the equilateral triangle formed by the moiré basis vectors is 5.1 nm at 1.6°, closely matching the orifice radius. Transition angles (θ_tr_) at different orifice radii are shown in Table 1. With a smaller radius, the crossover takes place at a larger angle as the moiré unit cell needs to reduce further and vice versa.

**Figure 3:**
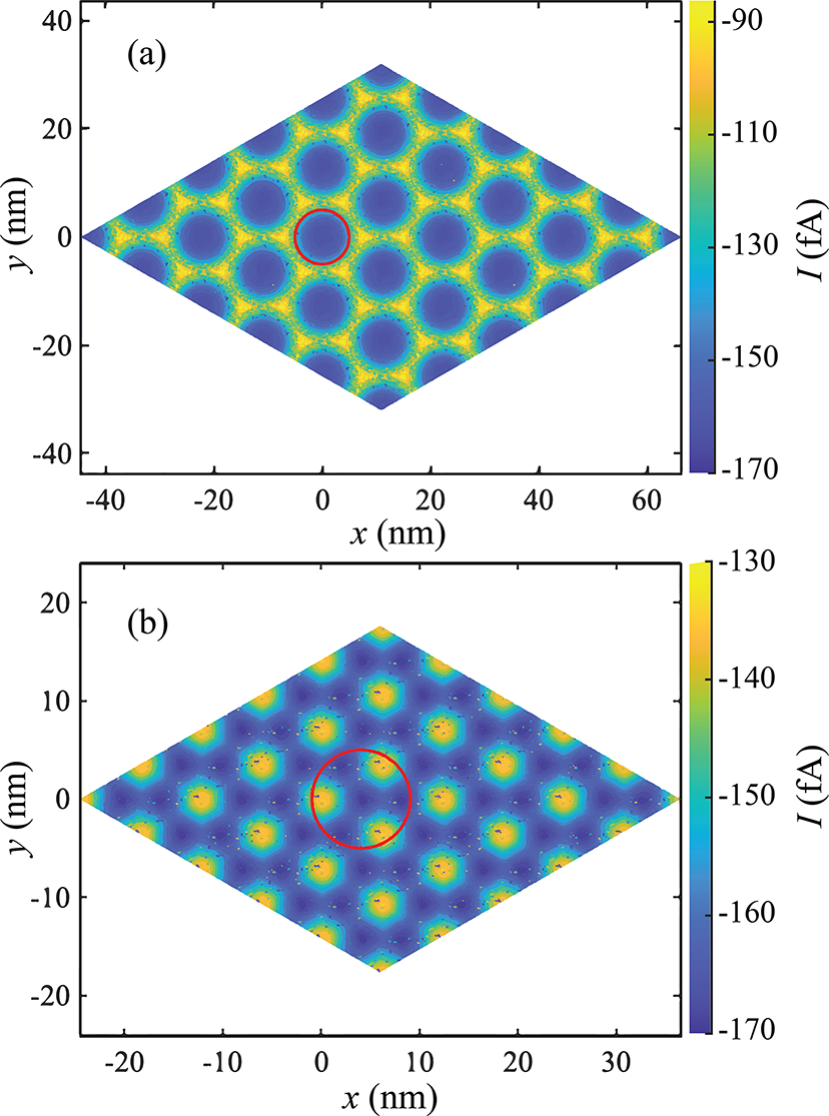
Current map calculated over a 3 × 3 moiré supercell for two twist angles, 1.1° (a) and 2° (b) at η = 0 V and a 5 nm orifice radius. Origin is set at the AA domain center. Current maximum (blue) is initially over the AA center in 1.1° (a), then switches to AB in 2° (b) twist. For reference, the orifice cross-section is marked (red circle) over AA in (a) and AB in (b), respectively.

Similarly, the current maxima will switch back to the AA center when the pipette radius reaches basis vector length. Theoretically, an infinite chain of AA/AB reversals takes place as the orifice radius is increased successively. However, the current resolution is only significant in fA until the first crossover from the AA to AB center. This sets a limit to the largest radius that can be employed effectively. In our case, the transition angle quickly reaches the magic angle (∼1°) for an 8 nm orifice radius (Table 1). Radii equal to and above 8 nm show negligible contrast between the two domains (Figure S2b). Due to this effect, the maximum current difference stays below 50 fA up to a 5 nm radius (Table 1).

Around the magic angle, the AA domain radius is 2.8 nm^70^ (Figure 2b), which allows orifice radii < 5 nm to be used. However, practical challenges arise in experiments along with a drop in mass transport limit that can interfere with domain currents. Table 1 summarizes current resolution values for different orifice radii. From our calculations, *a*_s_ = 2 nm is the lowest limit for effective current resolution between the domains, where the maximum current difference is 68% of its limiting current. Magnitude-wise, current difference is maximum (Δ*I*_m_ = 109 fA) at *a*_s_ = 3 nm, which matches expectation as it is closest to the AA domain radius (2.8 nm) at 1.1° twist. When *a*_s_ < 3 nm, the orifice area is smaller than the AA domain area, and when it is above 3 nm, weak current from AB spots increases in contribution. In both scenarios, the total current decreases when the nanopipette is centered on the AA spot. Figure 4a shows the SSV over AA/AB domains at 2 nm, where Δ*I*_m_ occurs at −0.05 V applied voltage (inset). At positive and negative voltage extremes, domain currents merge at zero (reaction limited) and at *I*_lim_ (mass transport limited), respectively. Hence, −0.05 V is the intermedial voltage where AA and AB domains are maximally distinct in their electrochemical response. From Table 1, the resolution ratio Δ*I*_m_/*I*_lim_ increases up to 2 nm. Below 2 nm, the mass transport limit is low, which obstructs the full current response at the AA domain. Additionally, practical challenges increase, including pipette fabrication, ICR, and droplet stability.

**Figure 4:**
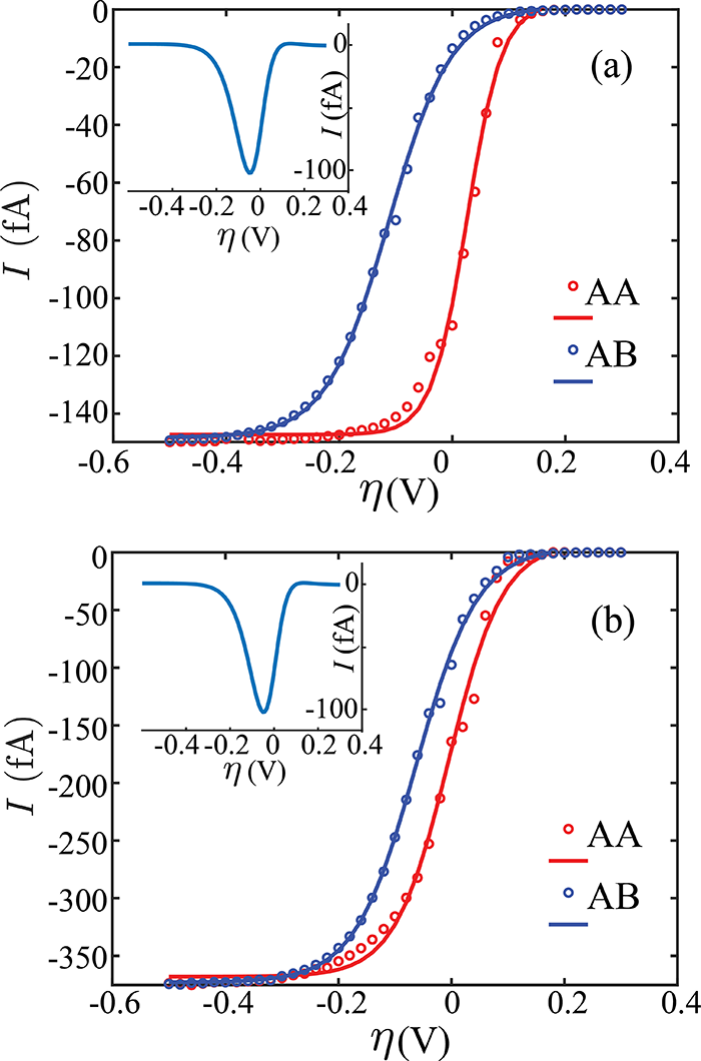
Simulated steady state voltammograms (dotted, fitted with sigmoids) on AA and AB domain centers at the magic angle (1.1°) using 2 nm (a) and 5 nm (b) orifice radii, respectively. The insets show the difference in the currents between AA and AB domains. The maximum current difference is −100.9 fA in both cases, occurring at −0.05 V. Ru^3+^ concentration is fixed at 2 mM at the top surface.

One way to magnify the current difference is to use a higher concentration of Ru^3+^ (>2 mM), which scales the current linearly but does not change the shape of the voltammogram. To ensure droplet stability, previous works mostly limit concentrations below 5 mM.^36,71^ In our case, the maximum current difference would increase to >250 fA if *c*_Ru^3+^_ = 5 mM. Another way to increase the resolution is to enable higher diffusion coefficients of the interacting redox couple. In Figure S4 and Table S1, we assume ∼3-fold lower diffusion coefficients (*D*_o_ = *D*_r_ = 3.7 × 10^–6^ cm^2^/s) corresponding to Co(phen_3_)^3+/2+^.^12^ Consequently, *I*_lim_ is 2.25× lower, and Δ*I*_m_ is 3× lower at ∼30 fA. Therefore, we anticipate that an increased diffusion coefficient correlates with a higher resolution ratio (Δ*I*_m_/*I*_lim_). Considering the nanostructure of tBLG, the orifice radii between 2 and 5 nm is most suitable for electrochemically observing the domains in both cases.

In conclusion, we have incorporated a high-dimensional kinetic rate model in simulation of the PNP equations, to investigate the electrochemistry of scanning a nanopipette over the tBLG surface. We solved the redox species concentration and flux at each coordinate by using local projected DOS within the modified MHC framework. Due to the localization of flat bands, the AA domain consistently exhibits a higher reaction rate and redox flux compared to any other region near the magic angle. This feature is lost at larger twist angles due to the diminished flat bands or with larger orifice radii, which average out the real-space variations. In addition, the location of current maxima fluctuates between AA and AB centers based on the number of AA domains enclosed by the orifice. On the other end, experimental viability is difficult with exceedingly small radii (∼1 nm), where the mass transport limit is low and hinders redox current from domains. Therefore, the two externally tunable parameters, orifice radius and twist angle, determine the limits of current resolution in twisted 2D materials. We identified a feasible range of orifice radii (2–5 nm) as having an optimal resolution with respect to its magnitude and limiting current. The current difference between the domains is maximum at an intermediate voltage (−0.05 V) in this range of radii. Under these conditions, the current difference (∼100 fA) is twice the magnitude of the manageable baseline noise (50 fA) in the SECCM setup, making it sufficiently large for a reasonable contrast between the domains. Hence, by employing an experimentally-informed model, we have assessed the limitations and resolution of SECCM over the tBLG system.

In the future, with our collaborators, we aim to fabricate small-sized nanopipettes on the order of a few nanometers and use them to verify the current results with a high signal-to-noise ratio. We shall attempt to diagnose the observed discrepancy between experiments and theoretical MHC-DOS predictions, likely due to twist dependence of the electron coupling term.^11^ Using this workflow, we can test empirical formulations of electron-coupling or other parameters using multivariate optimization techniques that fit experimental SSVs. Importantly, we can use the real-space electrochemical response from small nanopipettes to highlight the novel electronic properties in tBLG and other 2D materials. Prominent band structure features like VHS separation, peaks, and its spatial variations can be resolved electrochemically in flat-band systems.

## Supporting Information Available

(S1) Concentration map of Ru^3+^ species at the orifice for 1.1° and 4.6° twist angles, (S2) SSV over AA and AB domains at 4° twist and a 5 nm orifice radius and 1.0° twist and an 8 nm orifice radius, (S3) SSV and current map over a 3 × 3 moiré supercell at 1.6° twist and a 5 nm orifice radius; and (S4) SSV at 1.1° twist over 2 and 5 nm orifice radii assuming reduced diffusion coefficients corresponding to Co(phen_3_)^3+/2+^, whose summary of current differences is listed in Table S1 (PDF)
Transparent Peer Review report available (PDF)
