# Supplementary material for: Modeling Scanning Electrochemical Cell Microscopy (SECCM) in Twisted Bilayer Graphene
Source: J Phys Chem Lett. 2024 Jul 12;15(29):7371–8. doi: 10.1021/acs.jpclett.4c01002 (PMC11284846; doi:10.1021/acs.jpclett.4c01002)
Supplement: Supplementary file 1 — jz4c01002_si_001.pdf [file jz4c01002_si_001.pdf]

Supporting Information: Modeling Scanning Electrochemical Cell Microscopy (SECCM) in Twisted Bilayer Graphene

Mohammad Babar[†,‡] and Venkatasubramanian Viswanathan[*,¶,‡]

†*Department of Mechanical Engineering, University of Michigan, Ann Arbor, Michigan 48109, USA*

‡*Department of Mechanical Engineering, Carnegie Mellon University, Pittsburgh, Pennsylvania 15213, USA*

¶*Department of Aerospace Engineering, University of Michigan, Ann Arbor, Michigan 48109, USA*

E-mail: venkvis@umich.edu

Example solution of the Poisson Nernst Planck equations at the orifice of the 3D nanopipette is shown in fig. S1. $Ru^{3+}(NH_3)_6$ and supporting electrolyte (KCl) concentrations are fixed at 2 mM and 500 mM respectively at the top surface, as performed in an earlier work (see main text).

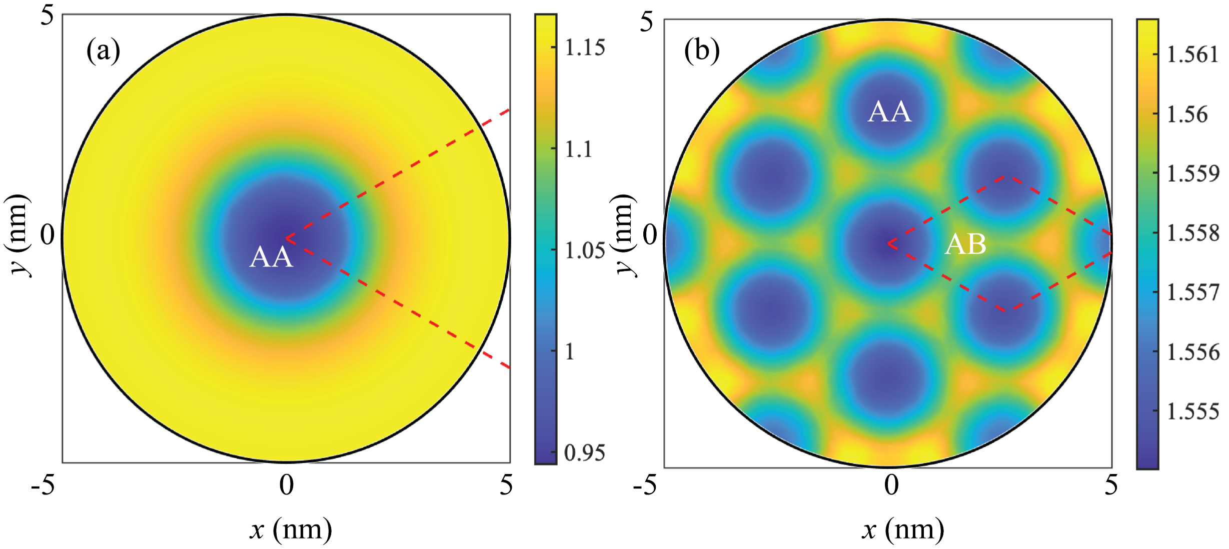

Figure S1: Concentration map (mM) of Ru^{3+} specie at the orifice (z=0) for two twist angles, 1.1° (a) and 4.6° (b) centered over the AA domain. Nanopipette radius is 5 nm and applied voltage is fixed at 0 V. Blue spots at AA domains are concentration minima and correspond to redox and ionic-flux maxima. Red boundary marks the moiré unit cell, which decreases monotonically with twist angle.

At large twist angles like 4° (Fig. S2a), steady state voltammogram is nearly identical between AA and AB centers due to diminished flat bands and small moiré unit cell size. Similarly, the resolution between AA/AB domains reduces with larger nanopipette radii. The last threshold is at 8 nm (Fig. S2b), where the transition angle (θ_{tr}, see main text) is at the magic angle (1.1°).

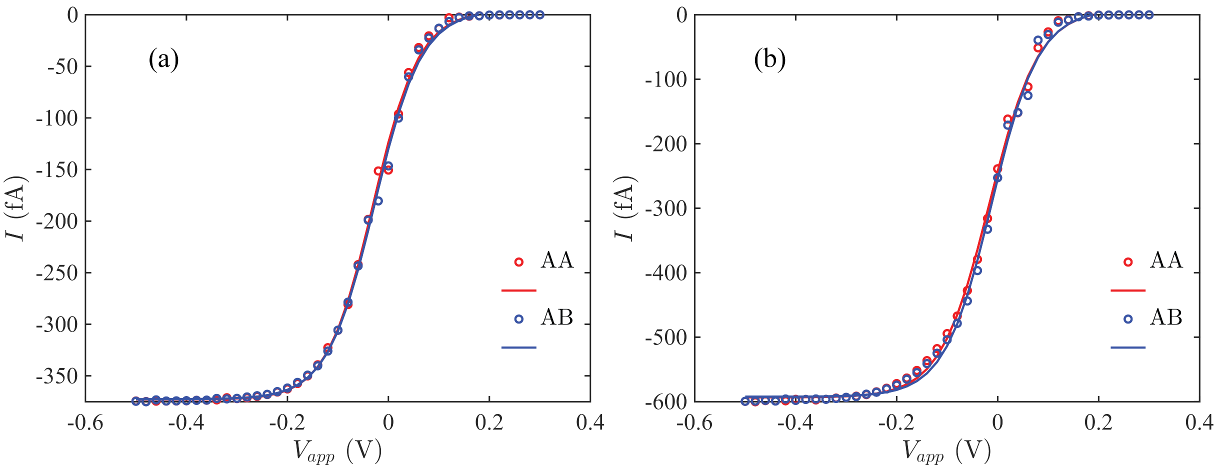

Figure S2: Steady state voltammogram at 4° twist and 5 nm radius (a), and 1.1° twist and 8 nm radius (b) with nanopipette centered at AA (red) and AB (blue) domains respectively. For both cases, the current difference between the domains is negligible. Ru^{3+} concentration is fixed at 2mM.

The transition angle θ_{tr} for 5 nm nanopipette radius lies at 1.6° twist, where the voltammograms are identical between AA/AB domains and current map exhibits minimal spatial variation (∼2 fA) across the supercell (Fig. S3). As shown in the figure, the current values in the 3×3 moiré contour are mostly green, i.e. localized around -144 fA (range ∼ 10 fA) at all spatial coordinates, which matches the corresponding value in the SSV marked with dashed lines and arrow. Hence there is zero resolution between the domains at this angle and orifice radius.

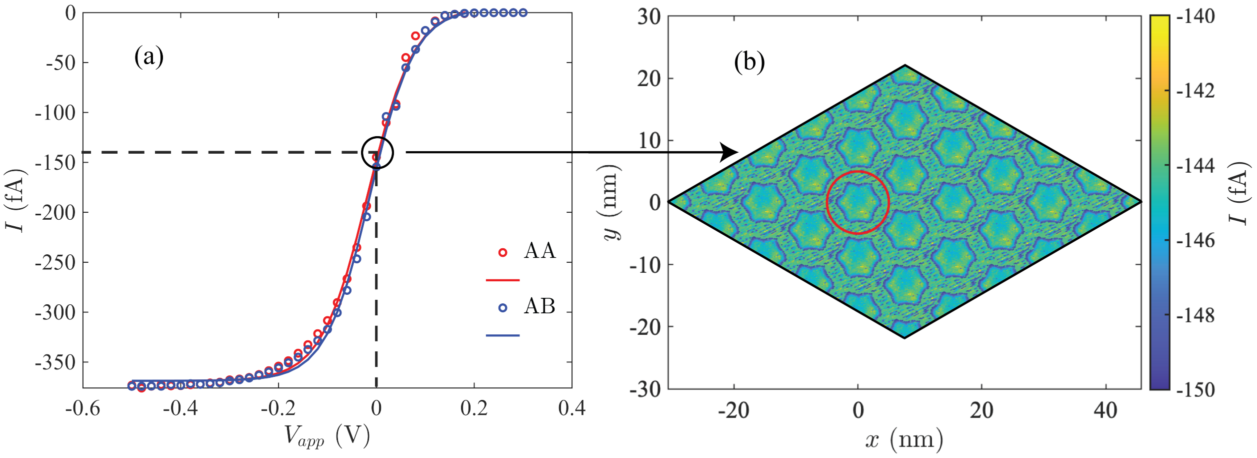

Figure S3: Steady state voltammogram of bilayer graphene twisted at 1.6° with 5 nm nanopipette radius (a). Current map of the same system over a 3×3 moiré supercell at equilibrium (η =0 V, marked with dashed lines and arrow). The current difference between AA/AB domains is minute (1.5 fA) at 0 V. The nanopipette radius (5 nm red circle) is marked over the AA center for reference. Ru^{3+} concentration is fixed at 2mM.

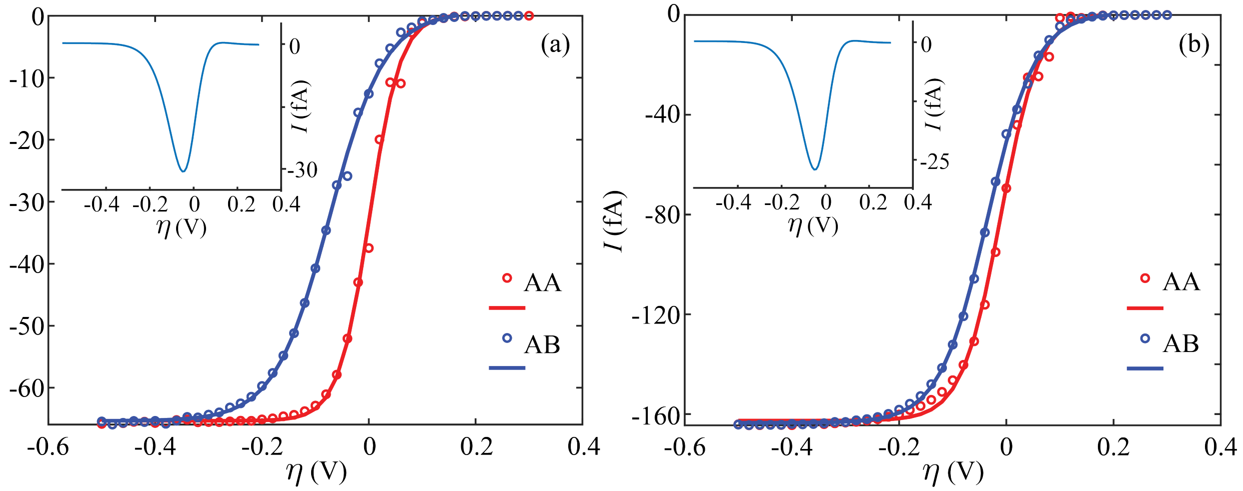

Figure S4: Simulated steady state voltammograms (dotted, fitted with sigmoids) assuming reduced diffusion coefficients corresponding to Co(phen$_3$)$^{3+/2+}$ redox couple ($D_o = D_r = 3.7 \times 10^{-6}$ cm^2/s) on AA and AB domain centers. The twist angle is fixed (magic angle, 1.1°) under 2 nm (a) and 5 nm (b) orifice radii respectively. The insets show the difference in the currents between AA and AB domains. The maximum current differences are -32.3 fA (a) and -26.5 fA (b), occurring at -0.05 V. Remaining parameters are same as in the main text.

Table S1: Summary of current resolution between domains with reduced diffusion coefficients corresponding to Co(phen$_3$)$^{3+/2+}$ redox couple ($D_o = D_r = 3.7 \times 10^{-6}$ cm^2/s). At each orifice radii (a_s, column 1), values for the transition angle (θ_{tr}, column 2) when current maxima switches from AA to AB center, circumradius (r_c, column 3) of the moiré basis vectors, maximum current difference ΔI_{m} at 1.1° twist (column 4), limiting current (I_{lim}, column 5) and resolution ratio ($\Delta I_{\mathrm{m}}/I_{\mathrm{lim}}$, column 6) are shown. Remaining parameters are same as in the main text.

a_s **(nm)**	θ_{tr} (°)	r_c **(nm)**	$\Delta I_{\mathbf{m}}$ **(fA)**	$I_{\mathbf{lim}}$ **(fA)**	$\Delta I_{\mathrm{m}}/I_{\mathrm{lim}}$
2	3.8	2.2	32.3	-65.5	0.49
3	2.6	3.1	32.4	-97.5	0.33
5	1.6	5.1	26.5	-162.6	0.16
8	1.0	8.2	0	-261.6	0
