# Supplementary material for: Modeling Scanning Electrochemical Cell Microscopy (SECCM) in Twisted Bilayer Graphene
Source: J Phys Chem Lett. 2024 Jul 12;15(29):7371–8. doi: 10.1021/acs.jpclett.4c01002 (PMC11284846; doi:10.1021/acs.jpclett.4c01002)
Supplement: Supplementary file 2 — jz4c01002_si_002.pdf [file jz4c01002_si_002.pdf]

jz-2024-010025.R1

Name: Peer Review Information for "Modeling Scanning Electrochemical Cell Microscopy (SECCM) in Twisted Bilayer Graphene"

First Round of Reviewer Comments

Reviewer: 1

Comments to the Author

The authors present here a theoretical study of a nanopipette approach, basically scanning electrochemical cell microscopy, to gain insight into the electrochemical behavior of twisted bilayer graphene, here using a twisted trilayer as a model. This manuscript seems to be a follow-up study of recent experimental work, in which the authors were involved (as cited in the manuscript, ref 10, 11) and at various occasions cited in this manuscript. The authors present here an advanced theoretical approach taking the Gerisher model for the distribution of the electronic states into account. The topic appears worth to be addressed and the paper may be published after following minor revisions.

The title is somewhat misleading, and it should be changed to reflect that this is a theoretical study.

Nanopipette-based scanning probe microscopy have been around now for at least two decades with significant hardware and software improvements in recent years. It appears that the authors avoid using the well-established term scanning electrochemical cell microscopy (SECCM) and instead refer to it as an "electrochemical microscope". Therefore, the authors should use SECCM rather than electrochemical microscope in order to avoid the impression that they want to claim novelty on an approach, which is well-established.

The authors should also comment on ionic current rectification effects, which may play a role in the described setup and experimental conditions.

The manuscript would really benefit from an experimental verification of the presented results. The authors state that this will be done in the near future, but again, even without the imaging approach but specifically local point measurements would be very interesting to support the data here.

Minor technical comments:

What is menat with “…the bottom surface of the nanopipette,..” the orifice of the nanopipette –also on page 3 (line 55) “…of the bottom surface…”

“RuHEX” is laboratory jargon and should be omitted in publications, so please use the correct formula $[Ru(NH_3)_6]^{3+}$ should be used.

Reviewer: 2

Comments to the Author

I had high hopes for this paper, based on the title and abstract, but was left very disappointed when I reviewed the content.

1. The abstract claims: "Here we introduce a “microscopy” technique…"

The technique described is SECCM (scanning electrochemical cell microscopy) now used by dozens of groups. The authors never mention this, rather describing the configuration in Fig 1 as though it were something new. This needs to be remedied with appropriate references and credit. Not least, without this, the work will be missed by the growing SECCM community. The authors are not introducing a new technique. Most disappointing the are no experiments, only very straightforward simulations. This makes the work borderline for JPC Lett.

2 Throughout, the authors need to make it clear this is a simulation paper - from the title to the abstract o the paper itself.

3.The authors talk of 'scanning' a single channel pipet with a tiny size. In fact, single channel SECCM has to work in a hopping mode, not a continuous scanning mode. With the latter, what would be the feedback signal? To determine the resolution needed and whether it is attainable requires analysis of errors that will be introduced from the back and forth motion of the pipet and avoiding overlap (or not) of the pipet spots. This is a slow process, so the authors would also need to estimate the time for a scan etc, and how that might impact the resolution (drift and other factors).

4. They also need to consider the noise level compared to signal that would be needed to land such a small pipet repeatably and reproducibly.

5. Pipet walls are charged. For this size pipet there will be significant current rectification. This has not been accounted for.

Author's Response to Peer Review Comments:

Response to Reviewers' Comments:

Real-Space Electrochemical "Microscopy" of Twisted Bilayer Graphene

Mohammad Babar[†,‡] and Venkatasubramanian Viswanathan[*,¶,‡]

†*Department of Mechanical Engineering, University of Michigan, Ann Arbor, Michigan 48109, USA*

‡*Department of Mechanical Engineering, Carnegie Mellon University, Pittsburgh, Pennsylvania 15213, USA*

¶*Department of Aerospace Engineering, University of Michigan, Ann Arbor, Michigan 48109, USA*

E-mail: venkvis@umich.edu

We thank the editor and reviewers for the reviews of our manuscript (ID: jz-2024-010025). We provide a point by point response to each of their comments. Reviewer comments are italicized and changes made in the manuscript are quoted in blue. We strongly believe that the revised manuscript should meet the standards for publication in the Journal of Physical Chemistry Letters (JPCL).

Before we proceed to respond to the reviewers, we want to specify a major change where we have corrected the diffusion coefficients for $Ru^{+3/+2}(NH_3)_6$ redox couple, i.e. D_o and D_r were amended to 8.43×10^{-6} cm^2/s and 1.19×10^{-5} cm^2/s respectively.[1] Our old values, D_o = D_r = 3.7×10^{-6} cm^2/s were erroneous as they corresponded to those of $Co(phen_3)^{3+/2+}$[2] redox couple. Subsequently, we redid our calculations and have updated the main text with new figures (3, 4 and S4). The revised voltammograms exhibit larger limiting currents and higher resolution between the domains, due to the ~ 3-fold increase in diffusion coefficients. We now have > 100 fA difference in the AA/AB currents, exceeding more than twice the noise threshold (50 fA) maintained by our experimental collaborators in their SECCM setup.[2] The effect of increased diffusion coefficient has now been specified in the main text and supplementary information.

Change (Paragraph 8):

Based on previous works,[1,2] we set D_o and D_r of $Ru^{+3/+2}(NH_3)_6$ to 8.43×10^{-6} cm^2/s and 1.19×10^{-5} cm^2/s respectively.

Change (Paragraph 19):

Another way to increase the resolution is to enable higher diffusion coefficients of the interacting redox couple. In figure S4 and table S1, we assume ~3-fold lower diffusion coefficients (D_o = D_r = 3.7×10^{-6} cm^2/s) corresponding to $Co(phen_3)^{3+/2+}$.[2] Consequently, I_{lim} is ×2.25 lower, and ΔI_m is ×3 lower at ~ 30 fA. Therefore, we anticipate that an increased diffusion coefficient correlates with a higher resolution ratio ($\Delta I_m/I_{lim}$).

Change (Paragraph 21):

Under these conditions, the current difference (~100 fA) is twice the magnitude of the manageable baseline noise (50 fA) in the SECCM setup, making it sufficiently large for a reasonable contrast between the domains. Hence, by

employing an experimentally-informed model, we have assessed the limitations and resolution of SECCM over the tBLG system.

---

1 Reviewer #1 Comments/Suggestions:

The authors present here a theoretical study of a nanopipette approach, basically scanning electrochemical cell microscopy, to gain insight into the electrochemical behavior of twisted bilayer graphene, here using a twisted trilayer as a model. This manuscript seems to be a follow-up study of recent experimental work, in which the authors were involved (as cited in the manuscript, ref 10, 11) and at various occasions cited in this manuscript. The authors present here an advanced theoretical approach taking the Gerisher model for the distribution of the electronic states into account. The topic appears worth to be addressed and the paper may be published after following minor revisions.

Author Reply: We thank the reviewer for their summary and helpful feedback. As described, we employ a SECCM setup and incorporate the Gerischer model into the PoissonNernst-Planck (PNP) simulations to determine the nanostructure-activity relationship in twisted bilayer graphene (tBLG). In the present work, we do not report results on the trilayer graphene due to the absence of an accurate momentum-space/tight-binding code that calculates its spatial DOS. We do have the total DOS model by Zhu et al.[3], which we employed in a recent work in JACS to map rate enhancement in twisted trilayer graphene. As pointed out correctly, this is a follow-up study of the recent experimental work, where we used the total DOS to observe highly enhanced reaction rates near the magic angle. Hopefully the results from this work allow an accurate estimation of the nanostructure rates based on the choosen nanopipette size and twist angle. We now respond to each of the reviewer's comments to the best of our abilities.

1. The title is somewhat misleading, and it should be changed to reflect that this is a theoretical study. Nanopipette-based scanning probe microscopy have been around now for at least two decades with significant hardware and software improvements in recent years. It appears that the authors avoid using the well-established term scanning electrochemical cell microscopy (SECCM) and instead refer to it as an ”electrochemical microscope”. Therefore, the authors should use SECCM rather than electrochemical microscope in order to avoid the impression that they want to claim novelty on an approach, which is well-established.

Author Reply: We appreciate the reviewer's comment and acknowledge our oversight. Accordingly, we have changed the title to indicate the theoretical study and modified the abstract and relevant paragraphs in introduction and conclusions to highlight the scanning electrochemical cell microscopy (SECCM) technique.

Change (Title):

Modeling Scanning Electrochemical Cell Microscopy (SECCM) in Twisted Bilayer Graphene

Change (Abstract):

Here we evaluate the nanostructure-activity relationship in twisted bilayer graphene by modelling it under the scanning electrochemical cell microscopy setup to resolve its spatial moir´e domains.

Change (Paragraph 2):

This correlation can be utilized to study the nanostructure-activity relationship in twisted graphene and to discern its spatial features through its electrochemical response. The scanning electrochemical cell microscopy (SECCM) is based on this working principle and has been widely studied for various systems like electrocatalytic and Li-ion cathode materials, aprotic solvents, nanoparticles, corrosion

at metal surfaces etc.[4–11]

Change (Paragraph 3):

The model has been tested in a SECCM setup to compare the activity of edge vs basal sites in graphene and graphite sheets.[12] Tuning the DOS for better overlap of the electrode states with redox couple transitions[13,14] has explained kinetic enhancement in systems like twisted layers of graphene,[2,15] single molecule reac-
tions with gold and copper,[16,17] lithium stripping and electrodeposition,[14] and reactivity of graphene edge states and defects.[18,19] Advances in fabrication of small pipette orifice diameters enables increased spatial resolution,[20] thus facilitating a real-space electrochemical study of the exotic electronic properties in twisted graphene.

Change (Paragraph 4):

In this letter, we shall use tBLG as a model system and theoretically evaluate resolution of the SECCM setup over its spatial domains. We shall derive a steady state solution of the ion-transport equations inside the nanopipette, as has been performed to simulate and fit voltammograms with experiments.[2,15] Using the finite element method, we solve for the electric potential and ionic concentrations self-consistently as defined by the Poisson-Nernst-Planck (PNP) equations. These equations have been utilized to report *i*-*V* curves in other systems.[21–24]

Change (Paragraph 5):

Figure 1 shows the schematic of the SECCM setup to be used for scanning the current at a range of overpotentials, as employed earlier on bilayer and trilayer graphene systems.[2,15]

Change (Caption Figure 1):

> Simplified schematic of the SECCM experimental setup where the nanopipette contains the redox couple ($Ru^{+3}(NH_3)_6$) and the supporting electrolyte (KCl) undergoing electron exchange with the substrate (tBLG) along the *z*-axis.

Change (Paragraph 20):

> Under these conditions, the current difference (~100 fA) is twice the magnitude of the manageable baseline noise (50 fA) in the SECCM setup, making it sufficiently large for a reasonable contrast between the domains. Hence, by employing an experimentally-informed model, we have assessed the limitations and resolution of SECCM over the tBLG system.

2. The authors should also comment on ionic current rectification effects, which may play a role in the described setup and experimental conditions.

Author Reply: The reviewer is correct in pointing out the missing ionic current rectification (ICR) effect. From previous works,[20,21] ICR is a result of the diffuse double layer (ddl) formed at the charged wall of the orifice, and can be minimized by increasing the supporting

electrolyte (here, KCl) concentration. Specifically with quartz nanopipettes used before,[2,15] ICR effect is significant if the KCl concentration is ≤ 100 mM for ~ 10 nm orifice radii.[20,22,25] Based on the expression given by Wei et al.[20] for quartz nanopipettes, if we increase the KCl conc. to 500 mM, 17% of the 5 nm orifice cross-section is covered by the ddl. High conc. of the supporting electrolyte also reduces ion transport from electromigration.[26] In our simulations, the voltammograms change only slightly when we increase the KCl conc. to 500 mM. This indicates that electromigration was already effectively shielded with the initial conc. of 100 mM. Increasing conc. by ×5 should significantly reduce non-linearity from ICR. Above 500 mM, our simulations encounter numerical instabilities, making them harder to converge and produce less consistent results.

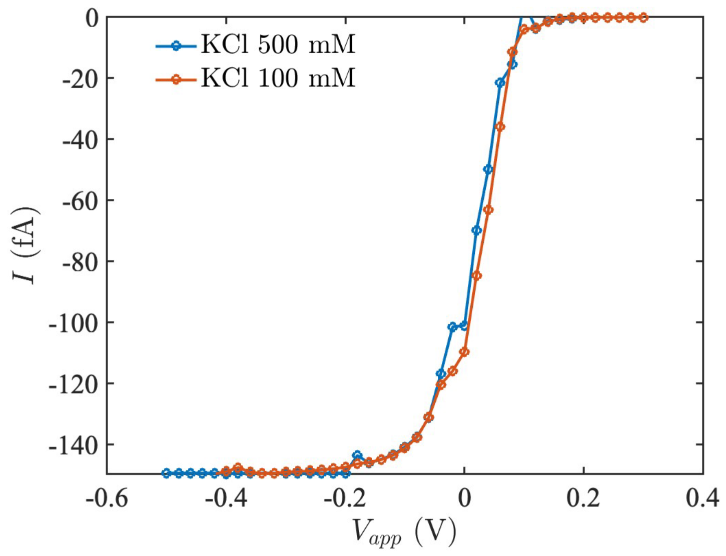

Figure 1: Comparison of simulated steady state voltammograms for (a) 100 mM and (b) 500 mM KCl concentrations. For both cases, a 2 nm radii nanopipette is centered at the AA spot and fixing 1.1 $^{\circ}$ twist angle in bilayer graphene.

We have changed KCl conc. to 500 mM and have specified these details in the main text.

Change (Paragraph 10):

A key difference from previous experiments is the concentration of the supporting electrolyte (KCl). The originally used concentration of 100 mM will induce substantial ionic current rectification (ICR) for orifice radii ∼5 nm in this study. Previous works[20,21] suggest that ICR is a result of the diffuse double layer (ddl) formed at the charged wall of the orifice, and can be minimized by increasing the concentration of the supporting electrolyte. Specifically with quartz nanopipettes used before,[2,15] ICR effect is significant if the KCl concentration is ≤ 100 mM for ∼ 10 nm orifice radii.[20,22,25] High concentration of the supporting electrolyte also reduces ion-transport from electromigration.[26] Hence to avoid a non-linear *i*–*V* response, we simulate the reactions with an increased KCl concentration of 500 mM, in which case the ddl occupies only 17% of the orifice cross-section.[20] This is the maximum value we

> can use without encountering numerical instabilities. If the measurements are stable, higher concentrations may as well be used in experiments.

3. The manuscript would really benefit from an experimental verification of the presented results. The authors state that this will be done in the near future, but again, even without the imaging approach but specifically local point measurements would be very interesting to support the data here.

Author Reply: We acknowledge the reviewer's suggestion and recognize the importance of experimental verification. However, we are limited by our collaborators in fabricating small-sized nanopipettes in the 2-5 nm range and in achieving low noise, which are necessary for local point measurements. We hope to follow up this study with a robust experimental analysis of SECCM on twisted graphene once we achieve proper nanopipette dimensions and noise reduction. We mention this limitation in the conclusions.

Change (Paragraph 21):

> In future, with our collaborators, we aim to fabricate small-sized nanopipettes on the order of a few nanometers and use them to verify the current results with high signal to noise ratio.

Though we mainly report simulation-based results in this work, we have high confidence in our predictions since the employed tight-binding method closely agrees with density functional theory (DFT) calculations, and the rate model prefactors have been trained with experimental values (figure 2(a) of the main text). For example, as shown in the figure below, Fang and Kaxiras[27] have demonstrated an excellent agreement between DFT and the tight-binding Hamiltonian for the twisted bilayer graphene. In the supplementary information of our recent work,[28] we derive a formula for uncertainty propagation in the Gerischer rates, which we find is <2% from the DOS.

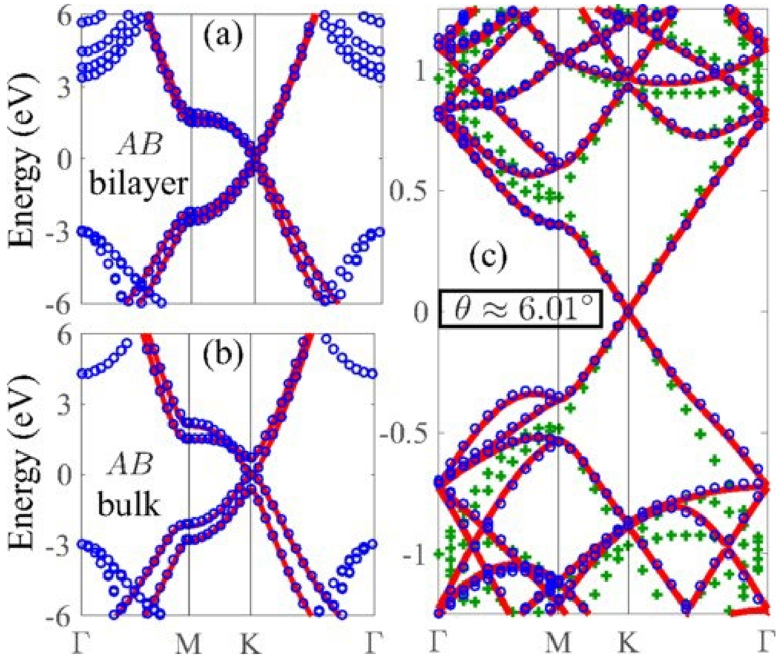

Figure 2: Comparison between the tight-binding Hamiltonian (red lines) and ab initio DFT (blue circles) band structure calculations. Reproduced from Fang and Kaxiras[27]. Copyright 2016 by the American Physical Society.

4. Minor technical comments: What is meant with "...the bottom surface of the nanopipette,.." the orifice of the nanopipette –also on page 3 (line 55) "...of the bottom surface..." **Author Reply:** We have replaced "the bottom surface" with "orifice" in the main text and in the supplementary information.

Change (Paragraph 4):

> Ionic flux and redox current are thus acquired at the orifice of the nanopipette, varying in response to the applied voltage, which alters the kinetic rates. The local electronic structure is not axi-symmetric as assumed before,[2,15] which requires simulation in a 3D nanopipette and evaluating the rates at each polar coordinate of the orifice.

Change (Paragraph 7):

However, local DOS is not axi-symmetric, hence to capture the spatial domains, we switched to the 3D mesh of the nanopipette where the reaction rates are unique at each polar coordinate of the orifice.

Change (Paragraph 8):

Eq. 2 forms the BC at the orifice, which is the only outlet for ionic flux.

Change (Paragraph 9):

The rest of the applied voltage drops across the electric double layer ($V_{dl} = \eta - V_q$), which is used to formulate a Robin-BC for electric potential ϕ at the orifice, based

on the Stern layer model,[29,30]

Change (Paragraph 12):

The applied voltage modifies the rate constants (Eq. 3), which on solving eq. 5,6 alters the Ru-ion flux (Eq. 2) and hence the current (Eq. 1) at the orifice.

Change (Paragraph 13):

An example solution of the described system can be visualized at the nanopipette orifice at two twist angles (1.1° and 4.6°, figure. S1).

5. "RuHEX" is laboratory jargon and should be omitted in publications, so please use the correct formula [Ru(NH3)6]3+ should be used.

Author Reply: We have changed these terms on recommendation of the reviewer.

Change (Paragraph 5):

The formal potential of the chosen redox couple, ruthenium hexamine ($Ru^{+3}(NH_3)_6$) in aqueous KCl, is closest to the charge neutrality point (CNP) of multilayer graphene,[12,15] allowing most efficient capture of flat bands and AA/AB domain resolution in measured currents.

Change (Caption Figure 1):

Simplified schematic of the SECCM experimental setup where the nanopipette contains the redox couple ($Ru^{+3}(NH_3)_6$) and the supporting electrolyte (KCl) undergoing electron exchange with the substrate (tBLG) along the *z*-axis.

2 Reviewer #2 Comments/Suggestions:

Recommendation: This paper may be publishable, but major revision is needed; I would like to be invited to review any future revision.

Comments: I had high hopes for this paper, based on the title and abstract, but was left very disappointed when I reviewed the content.

1. The abstract claims: "Here we introduce a "microscopy" technique.." The technique described is SECCM (scanning electrochemical cell microscopy) now used by dozens of groups. The authors never mention this, rather describing the configuration in Fig 1 as though it were something new. This needs to be remedied with appropriate references and credit. Not least, without this, the work will be missed by the growing SECCM community. The authors are not introducing a new technique.

Author Reply: We thank the reviewer for their comments and feedback, and we agree with the mentioned shortcomings. Accordingly, we have modified the abstract, figure caption and relevant paragraphs to highlight the scanning electrochemical cell microscopy (SECCM) technique.

Change (Abstract):

> Here we evaluate the nanostructure-activity relationship in twisted bilayer graphene by modelling it under the scanning electrochemical cell microscopy setup to resolve its spatial moir´e domains.

Change (Paragraph 2):

> This correlation can be utilized to study the nanostructure-activity relationship in twisted graphene and to discern its spatial features through its electrochemical response. The scanning electrochemical cell microscopy (SECCM) is based on this working principle and has been widely studied for

various systems like electrocatalytic and Li-ion cathode materials, aprotic solvents, nanoparticles, corrosion at metal surfaces etc.[4–11]

Change (Paragraph 3):

The model has been tested in a SECCM setup to compare the activity of edge vs basal sites in graphene and graphite sheets.[12] Tuning the DOS for better overlap of the electrode states with redox couple transitions[13,14] has explained kinetic enhancement in systems like twisted layers of graphene,[2,15] single molecule reactions with gold and copper,[16,17] lithium stripping and electrodeposition,[14] and reactivity of graphene edge states and defects.[18,19] Advances in fabrication of small pipette orifice diameters enables increased spatial resolution,[20] thus facilitating a real-space electrochemical study of the exotic electronic properties in twisted graphene.

Change (Paragraph 4):

In this letter, we shall use tBLG as a model system and theoretically evaluate resolution of the SECCM setup over its spatial domains. We shall derive a steady state solution of the ion-transport equations inside the nanopipette, as has been performed to simulate and fit voltammograms with experiments.[2,15] Using the finite element method, we solve for the electric potential and ionic concentrations self-consistently as defined by the Poisson-Nernst-Planck (PNP) equations. These equations have been utilized to report *i*-*V* curves in other systems.[21–24]

Change (Paragraph 5):

Figure 1 shows the schematic of the SECCM setup to be used for scanning the current at a range of overpotentials, as employed earlier on bilayer and trilayer

graphene systems.[2,15]

Change (Caption Figure 1):

Simplified schematic of the SECCM experimental setup where the nanopipette contains the redox couple ($Ru^{+3}(NH_3)_6$) and the supporting electrolyte (KCl) undergoing electron exchange with the substrate (tBLG) along the *z*-axis.

Change (Paragraph 20):

Under these conditions, the current difference (~100 fA) is twice the magnitude of the manageable baseline noise (50 fA) in the SECCM setup, making it sufficiently large for a reasonable contrast between the domains. Hence, by employing an experimentally-informed model, we have assessed the limitations and resolution of SECCM over the tBLG system.

2. Most disappointing is that there are no experiments, only very straightforward simulations. This makes the work borderline for JPC Lett. Throughout, the authors need to make it clear this is a simulation paper - from the title to the abstract to the paper itself.

Author Reply: We acknowledge the importance of experimental verification in this work. However, we are limited by our collaborators in fabricating small-sized nanopipettes in the 2-5 nm range and in achieving low noise in readings, which are necessary for local point measurements. We aim to follow up this study with a robust experimental analysis of SECCM on twisted graphene once we achieve proper nanopipette dimensions and noise reduction.

We mention this limitation in the conclusions.

Change (Paragraph 21):

In future, with our collaborators, we aim to fabricate small-sized nanopipettes on the order of a few nanometers and use them to verify the current results with high signal to noise ratio.

Though we mainly report simulation-based results in this work, we have high confidence in our predictions since the employed tight-binding method closely agrees with density functional theory (DFT) calculations, and the rate model prefactors have been trained with experimental values (figure 2(a) of the main text). For example, as shown in the figure below, Fang and Kaxiras[27] have demonstrated an excellent agreement between DFT and the tight-binding Hamiltonian for the twisted bilayer graphene. In the supplementary information of our recent work,[28] we derive a formula for uncertainty propagation in the Gerischer rates, which we find is <2% from the DOS.

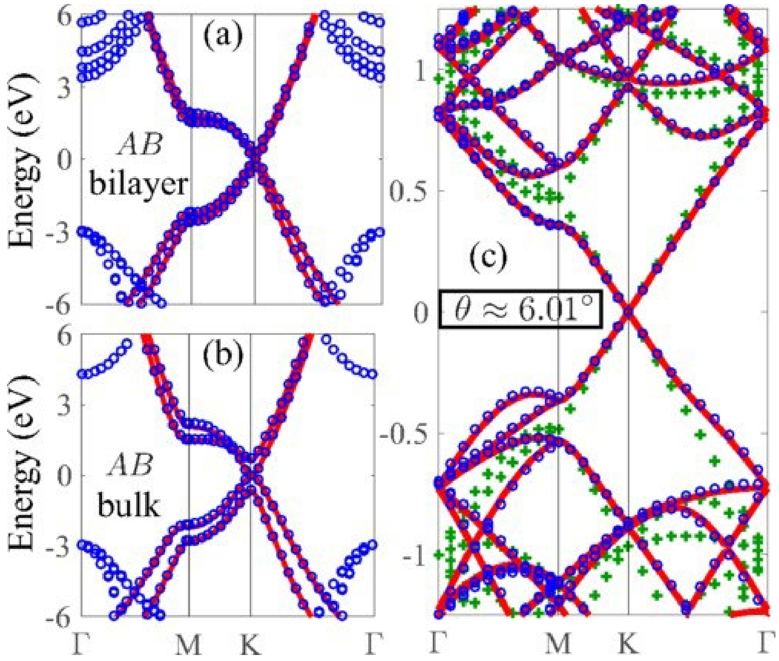

Figure 3: Comparison between the tight-binding Hamiltonian (red lines) and ab initio DFT (blue circles) band structure calculations. Reproduced from Fang and Kaxiras[27]. Copyright 2016 by the American Physical Society.

On recommendation of the reviewer, we have now modified the title, abstract and relevant paragraphs to emphasize that this is simulation-based study.

Change (Title):

Modeling Scanning Electrochemical Cell Microscopy (SECCM) in Twisted Bilayer Graphene

Change (Abstract):

Here we evaluate the nanostructure-activity relationship in twisted bilayer graphene by modelling it under the scanning electrochemical cell microscopy setup to resolve its spatial moir´e domains.

Change (Paragraph 4):

In this letter, we shall use tBLG as a model system and theoretically evaluate resolution of the SECCM setup over its spatial domains. We shall derive a steady state solution of the ion-transport equations inside the nanopipette, as has been performed to simulate and fit voltammograms with experiments.[2,15] Using the finite element method, we solve for the electric potential and ionic concentrations self-consistently as defined by the Poisson-Nernst-Planck (PNP) equations. These equations have been utilized to report *i*-*V* curves in other systems.[21–24]

Change (Paragraph 20):

Under these conditions, the current difference (∼100 fA) is twice the magnitude of the manageable baseline noise (50 fA) in the SECCM setup, making it sufficiently large for a reasonable contrast between the domains. Hence, by employing an experimentally-informed model, we have assessed the limitations and resolution of SECCM over the tBLG system.

3. The authors talk of "scanning" a single channel pipet with a tiny size. In fact, singlechannel SECCM has to work in a hopping mode, not a continuous scanning mode. With the latter, what would be the feedback signal? To determine the resolution needed and whether it is attainable requires analysis of errors that will be introduced from the back and forth motion of the pipet and avoiding overlap (or not) of the pipet spots. This is a slow

process, so the authors would also need to estimate the time for a scan etc, and how that might impact the resolution (drift and other factors).

4. They also need to consider the noise level compared to signal that would be needed to landsuch a small pipet repeatably and reproducibly.

Author Reply: We appreciate the reviewer's comments on the experimental noise introduced by operating in hopping mode and on the importance of achieving repeatable and reproducible measurements. Since this study is primarily simulation-based, our ability to comment on experimental technicalities is limited, therefore, we rely on our collaborators' SECCM setup to estimate the potential errors and other settings.[2,15] Our collaborators (Bediako group), who employed a 100 nm nanopipette to verify our previous predictions on twisted bilayer graphene,[15] ensured an overall baseline error of 50 fA in their setup before approaching or making final measurements. The optimal current difference between AA and AB domains is more than 2× (~100 fA) than this baseline error (see main text). Therefore, with 2-5 nm nanopipettes, we expect the SECCM setup to provide a reasonable resolution on tBLG. As correctly pointed out, a single channel SECCM has to work in a hopping mode. In figure 2(b) of our main text, we fitted the rate model prefactors with the experimental rate constants, which were measured in this hopping mode.[15] The back and forth motion does not contribute to further noise because the pipettes are allowed to stabilize for a minute after approaching and before measurement. To avoid overlapping between spots, in any mapping experiment, the pipette can hop for at least two pipette diameter distance between the two spots. The scan rate of hopping will depend on the wait time allowed for the pipettes to stabilize. Previously, the approach was set to be 0.2 μm/s and scanning potential at 100 mV/s. Drift can cause a loss in resolution, therefore the established drift correction techniques for microscopy can be employed.[31-33] We now mention these points in the main text for better readability and to support practical feasibility.

Change (Paragraph 5):

In the figure, a single channel SECCM will operate in a hopping mode. As we

describe later (figure 2(b)), the rate model prefactors were fitted on the experimentally measured rate constants, which were collected in hopping mode.[15] In our previous work,[15] we ensured an overall baseline error of 50 fA in our SECCM setup before approaching or making final measurements on tBLG. The pipettes can stabilize for a minute after approaching and before measurement to reduce additional noise from the back and forth motion. To avoid overlapping between spots, in any mapping experiment, the pipette can hop for at least two pipette diameter distance between the two spots. The scan rate of hopping will depend on the wait time allowed for the pipettes to stabilize. Previously,[2] the approach rate was set to be 0.2 μm/s and scanning potential at 100 mV/s. Drift can

cause a loss in resolution, therefore the established drift correction techniques for microscopy can be employed[31–33]

Change (Paragraph 20):

Under these conditions, the current difference (∼100 fA) is twice the magnitude of the manageable baseline noise (50 fA) in the SECCM setup, making it sufficiently large for a reasonable contrast between the domains. Hence, by employing an experimentally-informed model, we have assessed the limitations and resolution of SECCM over the tBLG system.

5. Pipet walls are charged. For this size pipette there will be significant current rectification. This has not been accounted for.

Author Reply: The reviewer is correct in pointing out the missing ionic current rectification (ICR) effect. From previous works,[20,21] ICR is a result of the diffuse double layer (ddl) formed at the charged wall of the orifice, and can be minimized by increasing the supporting

electrolyte (here, KCl) concentration. Specifically with quartz nanopipettes used before,[2,15] ICR effect is significant if the KCl concentration is ≤ 100 mM for ∼ 10 nm orifice radii.[20,22,25]

Based on the expression given by Wei et al.[20] for quartz nanopipettes, if we increase the KCl conc. to 500 mM, 17% of the 5 nm orifice cross-section is covered by the ddl. High conc. of the supporting electrolyte also reduces ion transport from electromigration.[26] In our simulations, the voltammograms undergo a small change when we increase the KCl conc. to 500 mM. This indicates that electromigration was already effectively shielded with the initial conc. of 100 mM. Increasing KCl conc. by ×5 should significantly reduce non-linearity from ICR. Above 500 mM, our simulations encounter numerical instabilities, making them harder to converge and produce less consistent results.

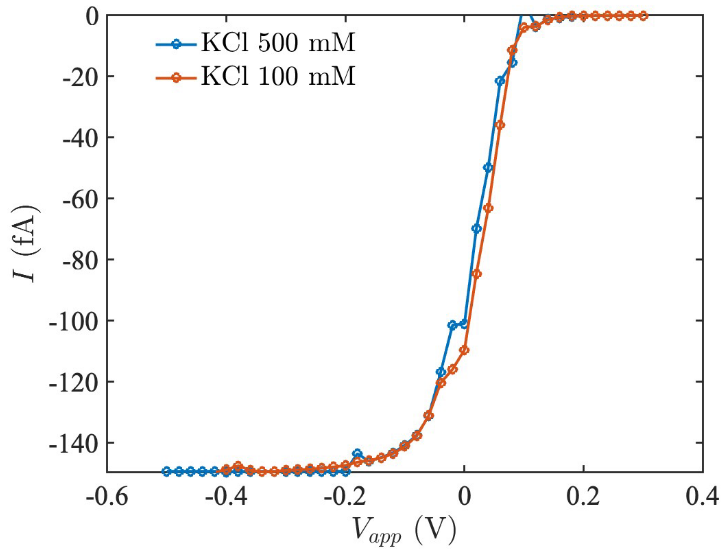

Figure 4: Comparison of simulated steady state voltammograms for (a) 100 mM and (b) 500 mM KCl concentrations. For both cases, a 2 nm radii nanopipette is centered at the AA spot and fixing 1.1 $^\circ$ twist angle in bilayer graphene.

We have changed KCl conc. to 500 mM and have specified these details in the main text.

Change (Paragraph 10):

A key difference from previous experiments is the concentration of the supporting electrolyte (KCl). The originally used concentration of 100 mM will induce substantial ionic current rectification (ICR) for orifice radii ∼5 nm in this study. Previous works[20,21] suggest that ICR is a result of the diffuse double layer (ddl) formed at the charged wall of the orifice, and can be minimized by

increasing the concentration of the supporting electrolyte. Specifically with quartz nanopipettes used before,[2,15] ICR effect is significant if the KCl concentration is ≤ 100 mM for ∼ 10 nm orifice radii.[20,22,25] High concentration of the supporting electrolyte also reduces ion-transport from electromigration.[26] Hence to avoid a non-linear i–V response, we simulate the reactions with an increased KCl concentration of 500 mM, in which case the ddl occupies only 17% of the orifice cross-section.[20] This is the maximum value we can use without encountering numerical instabilities. If the measurements are stable, higher concentrations may as well be used in experiments.

References

(1) Wang, Y.; Limon-Petersen, J. G.; Compton, R. G. Measurement of the diffusion coefficients of [Ru (NH3) 6] 3+ and [Ru (NH3) 6] 2+ in aqueous solution using microelectrode double potential step chronoamperometry. *Journal of Electroanalytical Chemistry* **2011**, *652*, 13–17.

(2) Zhang, K.; Yu, Y.; Carr, S.; Babar, M.; Zhu, Z.; Kim, B. J.; Groschner, C.; Khaloo, N.; Taniguchi, T.; Watanabe, K.; Viswanathan, V.; Bediako, D. K. Anomalous Interfacial Electron-Transfer Kinetics in Twisted Trilayer Graphene Caused by Layer-Specific Localization. *ACS Central Science* **2023**, *9*, 1119–1128.

(3) Zhu, Z.; Carr, S.; Massatt, D.; Luskin, M.; Kaxiras, E. Twisted trilayer graphene: A precisely tunable platform for correlated electrons. *Physical review letters* **2020**, *125*, 116404.

(4) Daviddi, E.; Gonos, K. L.; Colburn, A. W.; Bentley, C. L.; Unwin, P. R. Scanning electrochemical cell microscopy (SECCM) chronopotentiometry: development and applications in electroanalysis and electrocatalysis. *Analytical chemistry* **2019**, *91*, 9229–9237.

(5) Bentley, C. L.; Edmondson, J.; Meloni, G. N.; Perry, D.; Shkirskiy, V.; Unwin, P. R. Nanoscale electrochemical mapping. *Analytical chemistry* **2018**, *91*, 84–108.

(6) Bentley, C. L.; Unwin, P. R. Nanoscale electrochemical movies and synchronous topographical mapping of electrocatalytic materials. *Faraday Discussions* **2018**, *210*, 365– 379.

(7) Bentley, C. L.; Kang, M.; Unwin, P. R. Nanoscale structure dynamics within electrocatalytic materials. *Journal of the American Chemical Society* **2017**, *139*, 16813–16821.

(8) Bentley, C. L.; Kang, M.; Unwin, P. R. Scanning electrochemical cell microscopy

(SECCM) in aprotic solvents: Practical considerations and applications. *Analytical Chemistry* **2020**, *92*, 11673–11680.

(9) Bentley, C. L. Scanning electrochemical cell microscopy for the study of (nano) particle electrochemistry: From the sub-particle to ensemble level. *Electrochemical Science Advances* **2022**, *2*, e2100081.

(10) Yule, L. C.; Bentley, C. L.; West, G.; Shollock, B. A.; Unwin, P. R. Scanning electrochemical cell microscopy: A versatile method for highly localised corrosion related measurements on metal surfaces. *Electrochimica Acta* **2019**, *298*, 80–88.

(11) Takahashi, Y.; Kumatani, A.; Munakata, H.; Inomata, H.; Ito, K.; Ino, K.; Shiku, H.; Unwin, P. R.; Korchev, Y. E.; Kanamura, K.; others Nanoscale visualization of redox activity at lithium-ion battery cathodes. *Nature communications* **2014**, *5*, 5450.

(12) Guell, A. G.; Cuharuc, A. S.; Kim, Y.-R.; Zhang, G.; Tan, S.-y.; Ebejer, N.; Unwin, P. R. Redox-dependent spatially resolved electrochemistry at graphene and graphite step edges. *ACS nano* **2015**, *9*, 3558–3571.

(13) Royea, W. J.; Hamann, T. W.; Brunschwig, B. S.; Lewis, N. S. A comparison between interfacial electron-transfer rate constants at metallic and graphite electrodes. *The Journal of Physical Chemistry B* **2006**, *110*, 19433–19442.

(14) Kurchin, R.; Viswanathan, V. Marcus–Hush–Chidsey kinetics at electrode–electrolyte interfaces. *The Journal of Chemical Physics* **2020**, *153*.

(15) Yu, Y.; Zhang, K.; Parks, H.; Babar, M.; Carr, S.; Craig, I. M.; Van Winkle, M.; Lyssenko, A.; Taniguchi, T.; Watanabe, K.; others Tunable angle-dependent electrochemistry at twisted bilayer graphene with moir´e flat bands. *Nature chemistry* **2022**, *14*, 267–273.

(16) Boyen, H.-G.; Ziemann, P.; Wiedwald, U.; Ivanova, V.; Kolb, D. M.; Sakong, S.; Gross, A.; Romanyuk, A.; Bu¨ttner, M.; Oelhafen, P. Local density of states effects at the metal-molecule interfaces in a molecular device. *Nature materials* **2006**, *5*, 394– 399.

(17) Gu, M.-W.; Lai, C.-T.; Ni, I.-C.; Wu, C.-I.; Chen, C.-h. Increased Surface Density of States at the Fermi Level for Electron Transport Across Single-Molecule Junctions. *Angewandte Chemie* **2022**,

(18) Kislenko, V. A.; Pavlov, S. V.; Kislenko, S. A. Influence of defects in graphene on electron transfer kinetics: The role of the surface electronic structure. *Electrochimica Acta* **2020**, *341*, 136011.

(19) Pavlov, S. V.; Kislenko, V. A.; Kislenko, S. A. Fast method for calculating spatially resolved heterogeneous electron-transfer kinetics and its application to graphene with defects. *The Journal of Physical Chemistry C* **2020**, *124*, 18147–18155.

(20) Wei, C.; Bard, A. J.; Feldberg, S. W. Current rectification at quartz nanopipet electrodes. *Analytical Chemistry* **1997**, *69*, 4627–4633.

(21) Trivedi, M.; Nirmalkar, N. Ion transport and current rectification in a charged conical nanopore filled with viscoelastic fluids. *Scientific Reports* **2022**, *12*, 2547.

(22) Trivedi, M.; Gupta, R.; Nirmalkar, N. Electroosmotic transport and current rectification of viscoelastic electrolyte in a conical pore nanomembrane. *Journal of Membrane Science* **2022**, *659*, 120755.

(23) Tang, L.; Hao, Y.; Peng, L.; Liu, R.; Zhou, Y.; Li, J. Ion current rectification properties of non-Newtonian fluids in conical nanochannels. *Physical Chemistry Chemical Physics* **2024**, *26*, 2895–2906.

(24) Constantin, D.; Siwy, Z. S. Poisson-Nernst-Planck model of ion current rectification through a nanofluidic diode. *Physical Review E* **2007**, *76*, 041202.

(25) Smeets, R. M.; Keyser, U. F.; Krapf, D.; Wu, M.-Y.; Dekker, N. H.; Dekker, C. Salt dependence of ion transport and DNA translocation through solid-state nanopores. *Nano letters* **2006**, *6*, 89–95.

(26) Newman, J.; Balsara, N. P. *Electrochemical systems*; John Wiley & Sons, 2021.

(27) Fang, S.; Kaxiras, E. Electronic structure theory of weakly interacting bilayers. *Physical Review B* **2016**, *93*, 235153.

(28) Babar, M.; Zhu, Z.; Kurchin, R.; Kaxiras, E.; Viswanathan, V. Twisto-Electrochemical Activity Volcanoes in Trilayer Graphene. *Journal of American Chemical Society* **2024**,

(29) Stern, O. Zur theorie der elektrolytischen doppelschicht. *Zeitschrift fu¨r Elektrochemie und angewandte physikalische Chemie* **1924**, *30*, 508–516.

(30) Gongadze, E.; Petersen, S.; Beck, U.; Van Rienen, U. Classical Models of the Interface between an Electrode and an Electrolyte. COMSOL conference. 2009; pp 14–16.

(31) Liu, J. Scanning transmission electron microscopy and its application to the study of nanoparticles and nanoparticle systems. *Microscopy* **2005**, *54*, 251–278.

(32) Balinovic, A.; Albrecht, D.; Endesfelder, U. Spectrally red-shifted fluorescent fiducial markers for optimal drift correction in localization microscopy. *Journal of Physics D: Applied Physics* **2019**, *52*, 204002.

(33) Cnossen, J.; Cui, T. J.; Joo, C.; Smith, C. Drift correction in localization microscopy using entropy minimization. *Optics Express* **2021**, *29*, 27961–27974.

jz-2024-010025.R2

Name: Peer Review Information for "Modeling Scanning Electrochemical Cell Microscopy (SECCM) in Twisted Bilayer Graphene"

Second Round of Reviewer Comments

Reviewer: 2

Comments to the Author

The authors have taken on board the comments and the paper is better and a faithful representation of the work in the context of the field.

It was satisfying to see that common issues had been highlighted by the 2 reviewers and that the authors accepted the need to address these.

Author's Response to Peer Review Comments:

Response to Editors' Comments:

Modeling Scanning Electrochemical Cell Microscopy (SECCM) in Twisted Bilayer Graphene

---

Mohammad Babar[†,‡] and Venkatasubramanian Viswanathan[*,¶,‡]

†*Department of Mechanical Engineering, University of Michigan, Ann Arbor, Michigan 48109, USA*

‡*Department of Mechanical Engineering, Carnegie Mellon University, Pittsburgh, Pennsylvania 15213, USA*

¶*Department of Aerospace Engineering, University of Michigan, Ann Arbor, Michigan 48109, USA*

E-mail: venkvis@umich.edu

We thank the editor and reviewers for the positive response of our manuscript (ID: jz-2024010025). We provide a point by point response to each of the non-scientific changes

suggested by the editor. Editor comments are italicized and changes made in the manuscript are quoted in blue.

1. *Please move the TOC graphic to the correct position (on the same page as the abstract).*

Author Reply: Shifted the TOC graphic to the same page as the abstract.

2. *Please change all red and green text to black.*

Author Reply: The suggested changes have been made.

3. *One or more of your figures and tables includes a reference citation and permission documentation was uploaded. If any graphics were reproduced/adapted from previously published material, please provide the permission document (full license including terms and conditions) from the publisher and upload it as a "for editors only" file. Please also add a credit line in the caption in the following format: "Reproduced [or Adapted] from [reference number]. Copyright [YEAR] [Publisher Name]." The reference must be included in the references*

list.

Author Reply: To address the reviewers' comments, we had reproduced a graphic from a previous work, for which we had uploaded the mentioned permission document. The credit lines were also specified in our response to the reviewers. However, the adapted graphic is not included in either the main text or the supplementary information. We have now re-uploaded the permissions document under "Supporting Information for Review Only"

4. *Please add full publication information to references 6, 33, 39, and 65* **Author Reply:** The suggested changes have been made.

5. *Please submit your publication files without any markups. Please include annotated version(s) of your revised publication file(s) with colored text or highlights indicating the revisions that you have made as "Supporting Information for Review Only."* **Author Reply:** The suggested changes have been made.
